# Some Diseases of the Respiratory Tract from the View-point of the Pension Examiner1Read by invitation at the Lake Keuka Medical Association meeting, August 12, 1903.

**Published:** 1903-10

**Authors:** William Warren Potter

**Affiliations:** Buffalo, N. Y.


### Some Diseases of the Respiratory Tract from the View-point of the Pension Examiner.[1]

By WILLIAM WARREN POTTER, M. D., Buffalo, N. Y.

AMONG the commoner ailments of claimants for pension are "catarrh" and "disease of the lungs," these being the two general divisions which the civil war soldier makes of diseases of the respiratory tract. It is the common belief among old soldiers that a pneumonia contracted and suffered from in the service, has left behind it conditions which can be localised and differentiated even at this late day. Again, it is quite generally believed that pulmonary lesions, secondary to acute disease of the lower air tract suffered from in earlier life, remain as distinct factors of ill health, to be located, described, and rated forty years later.

### THE UPPER AIR TRACT.

The respiratory tract may be divided for convenience into two general topographical sections—upper and lower. The upper air passages extend from the nasal orifices to the larynx; all below that organ belong to the lower tract. In the upper area are situated the nasal cavities, the pharynx, and the larynx; in the lower are located the trachea, bronchial tubes, and air vesicles. The latter two groups are surrounded and held together by parenchymatous lung tissue, the whole constituting, in general terms, the lungs. We need not deal, at this time, with the pleura, because it is not, strictly speaking, a breathing organ.

Of all the diseases to which the upper air tract is liable, nasal catarrh is by far the most common. It afflicts all ages, conditions, and every environment of mankind, though some localities are remarkably free from it. Others, however, as for example, the North Atlantic seaboard and the great lakes region, are noted for its prevalence. While, therefore, it is not, strictly speaking, a climatic disease, yet climate exercises great influence over its general existence or severity—one or both.

It may be stated, in general, that moist atmospheres with sudden changes of temperature predispose to catarrhal diseases. It must not be forgotten, too, that rheumatic and gouty diatheses are underlying etiological factors of great moment in predisposing to catarrhal inflammations of the respiratory tract, especially in the nasopharynx, and this makes the subject one of additional interest to pension examiners.

1. Read by invitation at the Lake Keuka Medical Association meeting, August 12, 1903.

NASOPHARYNGEAL CATARRH.

However, before entering into details let us for a moment consider a few general features that lie at the threshold of the topic. In the first place there are many misconceptions relating to so-called nasal catarrh both among laity and physicians. The former quite generally believe that the disease leads ultimately to ulceration of soft parts and necrosis of bone; that it "runs into consumption;" that inflammation of the air tract is liable to be followed by similar conditions in all the cavities lined with mucous membrane, such as the stomach, intestinal and genitourinary tracts; in short, that all the mucous membranes are weak, nasal catarrh being an index of the fact. There is no just ground for such belief.

Physicians know that simple catarrhal inflammation of the nasal passages results only in hypertrophy of the tissues, whereas ulceration and necrosis belong to syphilis or some other systemic dyscrasia. While there is no doubt that chronic catarrhal inflammation tends to weaken tissue resistance and that it even may cause pharyngitis, laryngitis, or bronchitis, the disease, however, does not change its type and become tubercular in character. It has been asserted that a person with aggravated catarrhal disease of the upper air tract, and who has a family history of phthisis, is more liable to contract consumption than would be the case if the upper air tract were free from disease. I am not aware, however, that this asseveration has been sustained by any established clinical observations. So, while we may admit the tendency of catarrhal inflammation to travel downward, invading even the lower air passages, it still remains in its new location the same type of disease,—a true catarrhal affection.

It is, perhaps, unfortunate that a word which describes only a symptom should be so generally accepted as the name of a disease; and yet this is precisely what has happened with the word "catarrh." The Greek origin of the word signifies "to flow downward," *i. e.*, a discharge, and when we use the term nasal catarrh we mean that the person has a nasal discharge, acute or chronic, as the case may be. If we say a person has chronic nasopharyngeal catarrh, we mean he has a chronic catarrhal inflammation of the upper air tract.

The clinical picture of an ordinary catarrh may be stated as, first, a liability to take cold; second, swelling of the tissues and obstruction of the nasal air space; third, accumulation of thick mucus in the fauces. These are the three prominent factors all more or less intimately associated, and following each other in such rapid sequence, that it becomes difficult oftentimes, to locate the lesion. But it is highly probable that in most instances nasopharyngeal catarrh is a hypertrophic rhinitis, and that the latter in turn depends upon a deformed septum for its origin. Acute rhinitis, that is, acute inflammation of the mucous lining of the anterior nasal passages, is a disease of frequent occurrence, and when often repeated in a given case leads to the chronic form known as hypertrophied rhinitis. The location of the latter is most often the inferior turbinates, though the middle turbinates become similarly affected.

Since deformities of the nasal septum play such an important part in the causation of catarrhal diseases of the upper air tract, it becomes of interest to inquire for a moment into the etiology of these deviations. The weight of testimony would indicate that, first, traumatism plays an important part in producing septal deviations. I do not mean by this that there must needs have been a serious external blow, one, for example, hard enough to cause external deformity or fracture of the nose, or to visibly deflect or displace the septum. But, in young life, while the parts are soft and plastic, a fall on the face may cause a rupture of some of the sutural attachments of the septum, or push the cartilaginous septum against the vomer, or the perpendicular plate of the ethmoid, sufficiently to set up a mild form of inflammatory action. It is easy to conceive that from such a blow morbid processes may readily result in an angular deflection which, in a few years could and probably would cause limitation of air space and obstruction to breathing.

In a few years more, or even after the lapse of many years, new conditions arise, new environment takes place, new exposures are made, which singly or combined set up chronic hypertrophic inflammation of the mucous linings of the nares. It requires no stretch of the imagination to comprehend that such a morbid lesion is the result of a slow process of development, giving rise after many years to chronic catarrhal conditions, and that the original starting point is a slight injury received in childhood or infancy which attracted no attention whatever. The great point to be borne in mind is, that chronic nasal catarrh witnessed in adult life and old age, may be the result of a morbid process beginning in infancy in a traumatism so slight as to attract no attention, except that the child has "bumped his nose." This is entirely compatible with the history of disease and, in my view, accounts for the fact that so much chronic nasal disease is observed among old soldiers.

Again, in looking for causes we must not forget that the acute infectious diseases of childhood play an important rôle, etiologically speaking, in the chronic catarrhal diseases of the nasal passages of after-life. In a child that has already a deflected septum from causes just mentioned, measles, scarlet fever, diphtheria, and even typhoid fever, may leave as sequelæ the slow inflammatory processes that in later years cause serious nasal obstruction.

Still again, irritants such as exposure to cold, foreign bodies, hot air and steam, fumes of gases like chlorine, bromine, and ammonia, together with many other vapors, besides dust from elevators, mines, and factories, act with great direction and insistence upon nasal passages already prepared and made sensitive, by conditions set up in the manner described during early life.

That systemic diseases, such as gout and rheumatism and allied conditions, act as predisposing causes of nasal disease is an admitted fact, while age must be reckoned with in this relation, the resistance being greatest in adult middle life, and least in youth and old age.

Let me invite attention for a moment to the serious nature of the malady commonly designated as nasopharyngeal catarrh. Too often it is considered lightly, or as having no special importance,—little more in fact than a wart or a corn. It is, however, a disease of serious potentiality as every experienced physician knows. Obstruction of the nasal cavities often causes mouth breathing, which is a most unfortunate condition. This method of breathing not only renders the individual more liable to infection through the inspiration of germ-laden air but, likewise, it causes throat irritation, bronchial catarrh, and, indeed, may lead to serious pulmonary disease.

Stenosis of the nasal passages is one of the earlier symptoms of chronic nasal catarrh,—usually there being an alternating stenosis of the two passages. This latter phenomenon Bosworth believes to be pathognomonic of deflection of the septum. The effects of nasal obstruction if grouped would make a long list. Here are a few: (1) swelling or redness of the external nose; (2) intolerable itching of the nostril; (3) headache; (4) vertigo; (5) elongation of the uvula; (6) hypertrophy of the tonsils; (7) deformity of the chest; (8) spasmodic cough, asthma, and hay fever; (9) aphonia; (10) snoring; (11) constant or oft recurring catarrh of the pharynx, larynx, trachea, and bronchi; (12) perversion of the senses of smell and taste; (13) deafness; (14) eye affections; (15) stammering; (16) dyspepsia and gastralgia; (17) palpitation of the heart; (18) muscular rheumatism.

That the voice suffers changes in catarrhal disease is a fact of common observation. This in part is due to partial closure of the nasal chambers, and in other part to the laryngeal hyperemia secondarily set up by the catarrh. Various ear troubles are well known to arise from nasopharyngeal catarrh. Impaired hearing may develop either from nasal stenosis, or by extension of the inflammatory process to the tympanic cavity. Numerous neoplasms of the respiratory tract are attributable to nasal disease, and it is debatable also whether malignant growths may not arise from the same source, cases having been reported that tend to substantiate that view. Diseases of the accessory sinuses, too, frequently take their origin in obstructive nasal disease, drainage of these cavities thereby being interfered with. Finally, neuroses of the respiratory tract, as well as neuroses affecting other parts of the body, are sequelæ of nasopharyngeal disease,—an important fact to be remembered.

I have by no means exhausted the list of conditions and morbid changes, that have their beginning in catarrhal inflammations of the upper air tract, but enough have been enumerated to serve the purpose of this occasion, which chiefly is to point out the grave possibilities lurking in the train of what is called a "simple catarrh." In other words, there is, I asseverate, no such simplicity in this affection as is popularly believed, and too often is permitted to go undisputed by physicians.

There are obvious reasons why this discourse must limit itself to a cursory general view of the subject. It is, however, one that all examining surgeons are necessarily very much interested in, because they have to deal with it so frequently, either as a local condition or in its remote general effects. The old soldier suffering from the malady is entitled to great consideration. He is ignorant of its import, hence easily falls a prey to the horde of quacks whose methods appeal to his prejudices. He may have a simple form of the disease so far as its local manifestations are concerned; for example, one in which a slight operation may offer relief or cure, such as removal of a bony spur from the septum; or the snaring of an adenoid; or, again, the incising of an obstructive turbinate. But he is content to rely upon an advertised "catarrh cure," rather than to consult a laryngologist; indeed, his impecunious state is often a bar to an indulgence in that luxury. Moreover, often the gravest conditions cause the least distress; whereas, slight lesions may produce intense irritation. The first are neglected until great, perhaps irreparable damage is done; while the second are treated tentatively by incompetent persons. For these and other reasons not necessary here to enumerate, I repeat, the old soldier suffering from chronic affections of the upper air tract, is entitled to the best attention and most considerate judgment of the examining surgeon.

### THE LOWER AIR TRACT.

Passing now to a brief consideration of the diseases of the lower respiratory tract, we find the field somewhat limited if we are to consider only such as have their origin in the civil war service. The acute diseases are necessarily eliminated from the discussion as far as concerns pension examiners. Of the chronic forms phthisis pulmonalis is practically set aside as a primary affection, because those who contracted it in the military service during the war of 1861-5, are either cured or have gone to their reward, though there are many cases resultant from the Spanish War. It is, however, quite possible that occasional cases of tuberculous lung disease may yet be seen as secondary to some other form of affection either in the respiratory tract or other organs among Civil War soldiers. In my experience it is a disease rarely seen in the examining room of the pension surgeon. At all events, it is of easy recognition and comparatively of short duration. In its first stage it is susceptible of cure, but unfortunately it is not often seen early, and in its later stages it usually becomes fatal within a short time.

### CHRONIC BRONCHITIS.

A disease of frequent occurrence among old soldiers is chronic bronchitis, which is a catarrhal affection of middle or advanced life. It may appear as the result of repeated acute attacks, but more commonly as we see it, it is a primary disease assuming from the beginning a chronic type. Its development in most cases is gradual, extending over a period of years. Generally speaking, the symptoms are most marked in cold weather, and the "winter cough of the aged" is a form of the disease that is recognised by clinicians, as well as by the laity. Cough is, perhaps, the most troublesome symptom which unfortunately occurs chiefly at night, followed frequently by free expectoration in the early morning hours. It is associated many times with the gouty and rheumatic diatheses and occurs, too, in diabetic subjects, while it is common in both subacute and chronic forms in persons who drink vinous or alcoholic liquors. The presence of toxic elements in the blood seeking exit through the bronchi, serves to keep alive a chain of symptoms difficult to control, or impossible to cure.

It is essentially a disease of the larger bronchi, but there is always a tendency to exacerbations more or less acute, that may cause extension to the smaller tubes, and if they become obstructed serious results may ensue. Broncho-pneumonia, too, may result from further extension to the capillary bronchi. However, the most common sequelæ in long standing cases are pulmonary emphysema, and bronchiectasis, to which co-existing asthma may forcefully contribute. To these dangers must be further added the liability to dilatation of the cavities of the right heart with consequent pulmonary engorgement, congestion of the liver or other viscera, and lastly, edema of the extremities.

Chronic bronchitis is not classed, in itself, as a dangerous malady, at least not immediately so; but, indirectly, from its aggravating effects upon other disorders it may cause death from the complications it creates. Constant coughing, too, may further weaken a dilated and already laboring heart. Hemorrhages sometimes prove temporarily beneficial by relieving pulmonary congestion, but by and by, especially in feeble or aged persons, secondary manifestations may prove fatal. For these and other reasons chronic bronchitis is not to be regarded with indifference or as a slight affection in the old soldier. After secondary changes have taken place in the tubes it is incurable, hence treatment can only palliate. The tendency of rheumatic subjects to develop bronchitis is very marked, and such cases should be guarded against exposure with extreme care.

### ASTHMA.

One of the affections of the respiratory tract quite common among Civil War veterans is asthma. It is generally admitted to be a neurotic affection, characterised by spasm of the bronchial muscles and often associated with hyperemia of the bronchial mucous membrane. Since Hyde Salter's exhaustive treatise on asthma, published in 1860, many investigators have studied and written upon this fickle malady, yet none has disturbed his assertion of the neurotic character of the affection. Bosworth thinks it is a vasomotor bronchitis, and that the lesion in a paroxysm of asthma is a vasomotor paresis of the bloodvessels supplying the bronchial mucous membrane.

In my examination of soldiers I have been impressed with the frequency in which asthma was claimed, and with the comparative infrequency that physical signs of the malady were detected. I have been curious to learn whether the observation of other examiners corresponded with my own in this respect. I further observed that in all cases in which asthma was claimed and described there was intranasal disease of some variety, frequently associated, too, with bronchial catarrh.

In reflecting upon the subject I recalled a conversation with Bosworth held many years ago, in which be directed my attention to the relation of lesions of the nose and nasopharynx, to so-called asthma. I did not pay much heed to the subject at that time, but since the observations made in pension examinations it came back to me with much force. As this subject may be regarded as of some importance, I will quote from Bosworth's published writings somewhat in extenso.

About the time of our conversation it appears that Bosworth was preparing a paper on this very subject which later was published. In that monograph he assserted that "a large majority, if not all, cases of asthma are dependent upon some obstructive lesion in the nasal cavity. This is evidenced by the immediate relief from the exacerbation by the use of cocaine in the nose, in every case in which I have tried it, and, furthermore, by the cure of so many cases by the removal of the obstructive lesion in the upper air passages".

In another place, writing a few years afterward, Bosworth confirms this opinion, and further explains the fact, by pointing out the intimate relationship between the nose and lower respiratory tract, asserting that the respiratory functions of the nasal mucous membrane were overlooked until very recently; that even as late as 1878, Remy alluded to the nose as the organ of olfaction, asserting that its other functions are purely secondary and adventitious; that the great water-secreting function of the nasal mucous lining is not for phonation and olfaction, but for respiratory purposes; that the most intricate, most delicate, and most important part of the whole respiratory tract lies in the nose; that it resides in the turbinated tissues; that these bodies supply inspired air with moisture, pouring out upon their surfaces 16 ounces of water in the course of twenty-four hours; that in this way the inspired air becomes saturated with moisture, and that this function is regulated with such nicety, as to meet the changes from dry to moist atmospheres and the reverse, often sudden in character; all of which goes to establish a most intimate connection between the upper and lower portions of the respiratory tract. He then affirms that the blood supply of the nose being regulated by the same vasomotor tract as that which regulates the blood supply of the bronchial tubes, a disturbance in one region renders the other especially susceptible to disease processes, and that a diseased condition in the nasal cavity may predispose a neurotic person, under favorable atmospheric conditions, to an attack of asthma. I have not in this excerpt attempted to give the author's precise language, but offer merely a resume of his ideas. Other observers, writing at later periods, have confirmed Bosworth's studies.

From these premises, admitting their validity, which there is no reason to doubt, it must be affirmed as an established principle that intranasal disease, and especially when the nasopharynx is the seat of the lesion, is not only a predisposing but an exciting cause of paroxysms of asthma. True asthma is rarely associated primarily with cardiac disease, but the dyspnea attending the latter bears a resemblance to spasmodic asthma. Old soldiers almost universally complain of heart trouble, but comparatively few suffer from real disease of that organ. They often are afflicted with palpitation and other cardiac neuroses that arise from intranasal disease, and their asthmatic symptoms in general have a like origin. It becomes important to discriminate between idiopathic and pseudoasthma,—between the true disease and the reflex variety. This is not always easy, because there is nothing peculiarly pathognomonic in the symptoms or physical signs of nasal asthma, the paroxysms being identical with those caused by other lesions, except that preceding and after the attack the râles are dry, while in bronchial asthma they are moist.

CONCLUSIONS.

I do not speak from the standpoint of a laryngologist nor yet as an expert in diseases of the chest, hence cannot present the delicate points of the subjects of this discourse with the force and impact of the trained and skilful specialist in these branches; but I have aimed to give my own views based, perhaps, on no inconsiderable experience and observation, which have extended over a period of many years. I offer them to you for whatever value you may choose to place upon them, not dogmatically or with claims to original thought. Nevertheless, I shall have failed in my purpose if I have not accentuated a few important facts, with sufficient force for them to challenge consideration at your hands. They may be succinctly stated as follows:

First, that a person suffering with obstructive intranasal disease,—chronic nasopharyngeal catarrh,—is not laboring under a mere inconvenience, but has a malady that involves very serious danger, a danger that increases as years advance, and that, uncured, is a constant menace to the integrity of the lower respiratory tract.

Second, the neuroses dependent upon nasal obstruction are often as serious in their symptomatology, as they are distressing to the patient. They affect not only the respiratory tract, but the heart and other organs, often masking the real disease to an extent that may embarrass the examiner in making a differential diagnosis.

Third, that chronic catarrhal bronchitis is a disease of potential importance, slow in its progress, often depending upon nasal obstruction as a primary factor of cause, and is of most serious import in the aged.

Fourth, that asthma is, for the most part, a reflex manifestation of nasopharyngeal disease, generally obstructive in character; that it is seldom idiopathic, but its paroxysms are similar whether it be true or false, whether it be reflex or bronchial; and that its chief importance is as a symptom of disease located elsewhere, spasm of the bronchial tubes being a distressing expression of the lesion.

Fifth, that those diseases of the respiratory tract to which the veteran soldier is peculiarly liable, are often expressions of the rheumatic or gouty diathesis, and that these dyscrasiæ are often underlying factors to be reckoned with in reference to origin as logical sequences of pathology.

As a final thought, permit me to suggest that it does not require much mental elasticity or any distortion of facts, to affirm that a Civil War veteran suffering from chronic catarrhal disease of the respiratory tract, is as much entitled to rating and pension as though he were shot in battle. Such a man who, in childhood, received a slight injury to the nose, causing deformity of the septum, and in young manhood through exposure in the military service, contracted intranasal disease which later, after the development of rheumatism, appears as chronic rhinitis, bronchial catarrh, or pseudoasthma, is entitled to serious and considerate treatment at the hands of the government.

For these and other reasons that need not be multiplied, it becomes of the utmost importance for examining surgeons to give becoming weight to these maladies and their ratings. There is abundant reason to believe that the pension bureau desires the most careful consideration of the claims of the veteran at our hands, to the end that his interests be safeguarded on the one hand, and that the government be protected from imposition on the other; and especially does it desire to be generous rather than niggardly, in dealing out its largess to the defenders of the nation's honor. It is in this spirit that I have presented this brief paper.

284 Franklin Street.

